# DISCHARGE PREPARATION FOR OLDER ADULT IN PROLONGED EMERGENCY: A CASE-CONTROL STUDY

**DOI:** 10.1093/geroni/igae098.3090

**Published:** 2024-12-31

**Authors:** Jinming Liu, Fengli Gao, Zijing Chen

**Affiliations:** Beijing Tsinghua Changgeng Hospital, Beijing, Beijing, China (People’s Republic); Beijing Tsinghua Changgeng Hospital, Beijing, Beijing, China (People’s Republic); The First Hospital Of Jilin University, Changchun, Jilin, China (People’s Republic)

## Abstract

This study aims to explore the effectiveness of discharge preparation services in older adult with prolonged emergency observation and functional impairment. From August 2020 to June 2023, older adult (age ≥60 years) with Barthel Index < 100 and a hospital stay of >7 days in the emergency observation ward at a tertiary hospital in Beijing, China were included. A retrospective case-control study was conducted. The intervention group, guided by dedicated nurses, underwent personalized assessments, comprehensive care planning, and follow-up, while the control group received standard care. Primary outcomes included average hospitalization costs, length of stay, and 30-day readmission rates. A total of 628 patients were included after one-to-one nearest-neighbor matching without replacement. Among them, 314 received routine discharge preparation, and 314 received specialized nurse-led discharge preparation. Both groups demonstrated no statistically significant differences in baseline characteristics. The intervention group showed no significant difference in average hospitalization costs (72030 ± 63533RMB vs. 72446±68282RMB, t = 1.99, P = 0.906) or average length of stay (36 ±32days vs. 36 ±27days, t = 1.159, P=0.803). However, the intervention group exhibited a significantly lower 30-day readmission rate compared to the control group (6.7% vs. 15%, t = 3.36, P = 0.008). The findings suggest that while discharge preparation services may not impact immediate costs or length of stay for older adult with prolonged emergency observation and functional impairment, they significantly contribute to reducing the 30-day readmission rate. This emphasizes the importance of specialized nurse-led interventions in enhancing post-discharge outcomes and reduce healthcare system burden.

